# Abdominopelvic hydatid disease during pregnancy: a systematic review of the literature

**DOI:** 10.3389/fgwh.2026.1928979

**Published:** 2026-08-27

**Authors:** Evangelos Chinis, Kyriakos Tzanakis, George Lockett, Laura Burney Ellis, Stergios Bobotis, Georgios Geropoulos, Maria Kyrgiou, Konstantinos S. Kechagias

**Affiliations:** 1 Department of Surgery, School of Medicine, National and Kapodistrian University of Athens, Athens, Greece; 2 Department of Metabolism, Digestion and Reproduction, Faculty of Medicine, Imperial College London, London, United Kingdom; 3 Department of Obstetrics and Gynaecology, Imperial College Healthcare NHS Trust, London, United Kingdom; 4 Department of General Surgery, Maidstone and Tunbridge Wells NHS Foundation Trust, Maidstone, United Kingdom; 5 Department of General Surgery, Addenbrooke’s Hospital, Cambridge University Hospitals NHS Foundation Trust, Cambridge, United Kingdom

**Keywords:** echinococcal cyst, infectious disease, liver, pregnancy, pregnancy complication

## Abstract

**Objective:**

To review the available literature on abdominopelvic cystic echinococcosis (CE) during pregnancy, focusing on clinical presentation, diagnostic approaches, management strategies, and maternal and foetal outcomes.

**Methods:**

A systematic review was conducted in accordance with PRISMA guidelines. Studies reporting abdominopelvic hydatid disease in pregnant women were included without restrictions on study design or geography.

**Results:**

Fifty-one studies (45 case reports and 6 case series) including 79 patients were analysed. The mean maternal age was 28 years (range 15–42). Most patients with reported clinical-presentation data were symptomatic (56/67, 83.6%), commonly presenting with right upper quadrant or nonspecific abdominal pain. Ultrasound was the primary diagnostic modality (50/52, 96.1%), while MRI was used in selected cases. The liver was the most frequently involved site (50/72, 69.4%), followed by pelvic and adnexal locations. Surgical intervention was performed in 38.5% of cases, conservative management in 30.8%, and cyst aspiration in 14.1%. Surgery was often timed during caesarean section or the second trimester. Benzimidazole therapy was administered to 31 patients, most commonly during pregnancy. Caesarean delivery occurred in 43.3% and vaginal delivery in 34.3% of cases. Adverse pregnancy outcomes included miscarriage (4.5%), termination of pregnancy (3.0%), one maternal death (1.5%), and one neonatal death (1.5%). Among the 36 pregnancies with reported gestational age at delivery, 13 (36.1%) resulted in preterm birth.

**Conclusion:**

Abdominopelvic CE during pregnancy is rare and clinically challenging, with substantial heterogeneity in presentation and management across the published literature. Given the limited available evidence, management should be individualised within a multidisciplinary team, while prospective international registries are needed to strengthen the evidence base.

## Introduction

Human echinococcosis is a parasitic zoonotic disease (1). Echinococcus granulosus is the causative pathogen of cystic echinococcosis (CE), a rare condition also known as hydatid disease (1). CE is prevalent in rural areas where shepherding practices are common (1). Humans may become infected either through contact with contaminated animals or through the consumption of food or water containing parasite oocysts (2). The reported incidence of the disease during pregnancy is approximately 1 in 20,000 (3). Clinical manifestations depend on cyst size and location. Pregnancy-related immunological changes and placental steroids may promote cyst enlargement, likely reflecting accelerated parasite growth (4).

The liver and lungs are most commonly affected, though other abdominopelvic organs may also be involved (5). The incidence of pelvic hydatid cysts ranges from 0.2% to 2.25%, with nearly 80% located in the ovaries or uterus (6). Other possible abdominal sites include the spleen, kidney, pancreas, and peritoneum (7, 8). Hydatid disease may mimic other pathological conditions and, depending on the anatomical site or in the event of cyst rupture, may present as an acute abdomen (9, 10). Although rupture is rare, it is particularly dangerous, as it may facilitate a cascade leading to anaphylaxis (3). Cysts located in the abdominopelvic cavity may pose a risk to maternal and foetal safety by increasing the risk of rupture, preterm labour, and miscarriage (4, 11). In many cases, cysts are diagnosed incidentally during routine pregnancy imaging (12). The need to limit ionising-radiation exposure during pregnancy may further complicate diagnostic assessment (13).

Management of hydatid disease in pregnancy is challenging, as medical therapies carry teratogenic risks and surgical intervention is associated with adverse maternal and foetal outcomes, with optimal timing remaining uncertain (14). To date, no evidence-based guidelines providing standardised recommendations for the diagnosis and management of CE during pregnancy have been published (15). This systematic review summarises the literature on abdominopelvic hydatid disease in pregnancy, including presentation, diagnosis, management, and outcomes, to support clinical decision-making and guide practice.

## Methods

This review was reported in accordance with the Preferred Reporting Items for Systematic Reviews and Meta-Analyses (PRISMA) guidelines. The study protocol was prospectively registered with PROSPERO (Registration No. CRD420251232491).

### Literature search

Two reviewers (EC and KT) independently searched PubMed/MEDLINE, Ovid, and Scopus from database inception to November 2025. The search combined terms relating to cystic echinococcosis or hydatid disease with terms relating to pregnancy: (Echinococcosis OR hydatid disease OR hydatid cyst) AND (pregnancy OR pregnant), adapted to the syntax of each database. The searches retrieved 264 records from PubMed/MEDLINE, 668 from Ovid, and 399 from Scopus before deduplication. No restrictions according to study design or geographic region were applied at the search stage. Reference lists of included reports and relevant reviews were also searched manually. Disagreements were resolved by a third investigator (KSK).

### Eligibility criteria

Studies reporting abdominopelvic hydatid disease in pregnant women were eligible for inclusion, and all study designs were considered. Only full-text articles published in English were included. Reports were excluded when it was unclear whether symptoms were attributable to echinococcosis or when the disease was diagnosed only after pregnancy. Review articles, conference abstracts, and non-peer-reviewed sources were also excluded.

### Data extraction and handling

Two authors (EC and KT) independently extracted patient-level data from all included reports. Discrepancies were resolved by a third reviewer (KSK), with any remaining disagreements discussed with a fourth author (GG). Extracted variables included publication year, study design, age, ethnicity, parity, trimester at diagnosis, previous hydatid disease, diagnostic assessment, presenting symptoms, laboratory findings, therapeutic plan during pregnancy, surgical management, cyst location and number, cyst size on ultrasound, timing of intervention, pregnancy outcomes (including foetal and maternal outcomes when available), follow-up findings, postoperative imaging, serology results, follow-up duration, operative approach (open, laparoscopic, or robotic), and the use and timing of antiparasitic therapy.

### Quality assessment

All included studies were case reports or case series with individual patient data. Methodological quality was evaluated using the Joanna Briggs Institute (JBI) critical appraisal checklists. Two authors (EC and KT) independently performed the assessments. For case reports, the assessed domains were patient demographics, medical history, clinical presentation, diagnostic tests, intervention(s), post-intervention condition, adverse or unanticipated events, and takeaway lessons. Case series were assessed using the corresponding JBI domains for inclusion criteria, condition measurement and identification, consecutive and complete inclusion, demographics, clinical information, outcomes or follow-up, presenting sites, and statistical analysis. Each domain was rated as “Yes,” “No,” “Unclear,” or “Not Applicable” (Supplementary Tables S1, S2).

### Data synthesis

This review used descriptive statistics to summarise demographic and clinical characteristics, including presenting symptoms, imaging methods, treatment approaches, follow-up duration, and outcomes. Continuous variables are summarised using means and ranges, while categorical variables are presented as counts and percentages. Denominators reflect the number of cases with data available for each outcome and therefore vary across analyses. No comparative meta-analysis was performed because the evidence consisted predominantly of case reports and small case series with substantial clinical and reporting heterogeneity.

## Results

**Figure 1:**
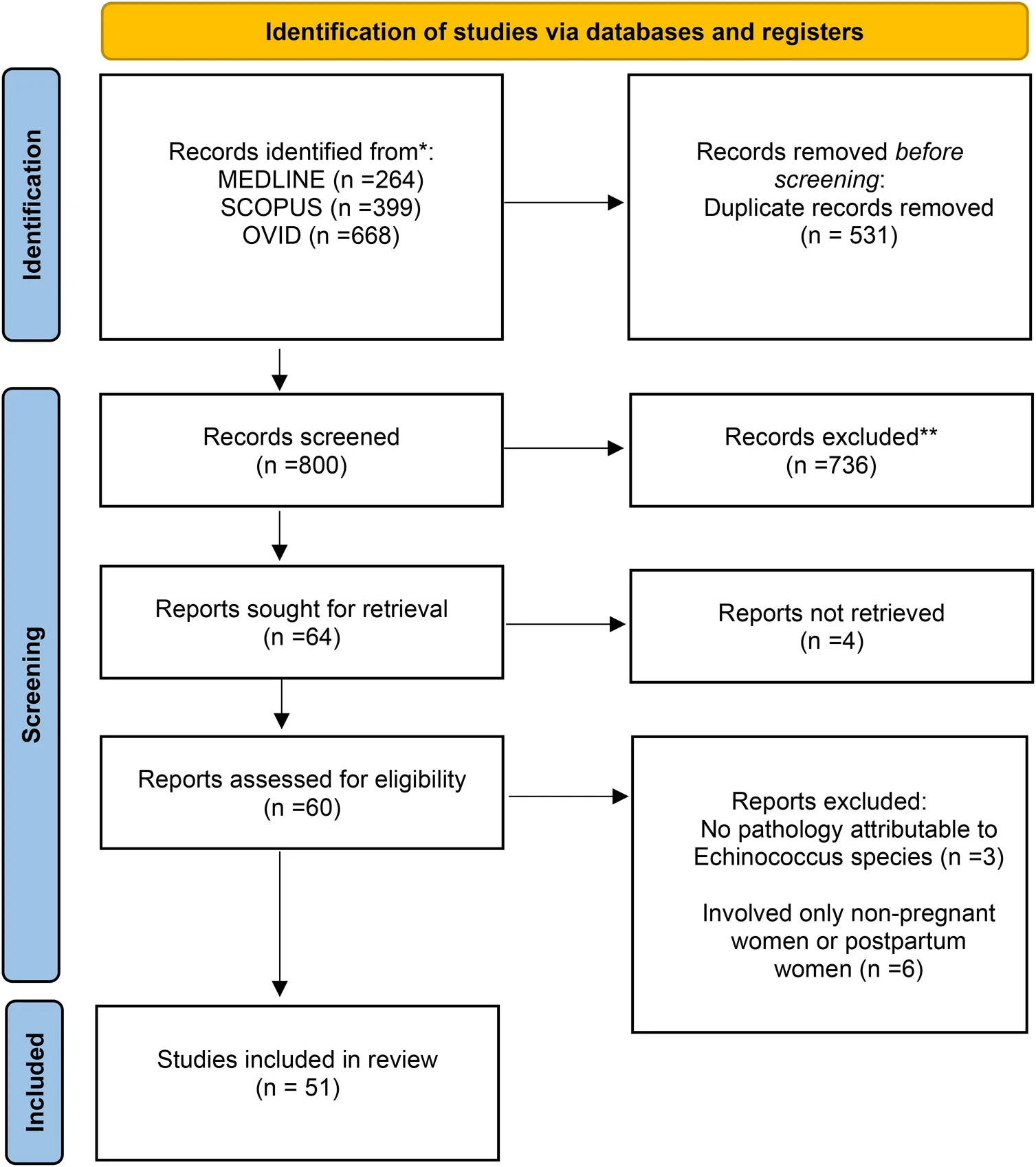
PRISMA flow diagram of our systematic review.

The search identified 1,331 records, of which 531 were duplicates. During title and abstract screening, 736 records were excluded, and 64 reports were sought for retrieval. Four reports could not be retrieved, leaving 60 reports for full-text assessment. Nine reports were excluded: three did not describe pathology attributable to Echinococcus species, and six included only non-pregnant or postpartum women. Ultimately, 51 publications were included in the review (Figure 1) (2, 11, 13–61). In total, 45 studies were case reports (Table 1), while six were case series (Table 2).

**Table 1:** Characteristics of the included studies (case reports).

Author, year	Case number	Country	Age	Trimester of diagnosis	Presenting symptoms	Diagnostic modality	Cyst location	Number of cysts	Pregnancy outcome	Therapeutic management	Trimester/timing of management
Steriu et al. (2)	Case 1	Romania	29 yo	2	Abdominal discomfort, fever	U/S, laboratory workup, serology Ech., vaginal culture, urinalysis	Liver	Multiple	CS, 38-39 GW	Surgery	2
Maheshwari et al. (16)	Case 1	India	early 30s	3	Asymptomatic/incidental finding	U/S, laboratory workup, pelvic MRI	Peritoneum	Multiple	CS, 32 GW	Spontaneous labor & surgery	During CS
Fanning (11)	Case 1	USA	20 yo	N/A	Asymptomatic/incidental finding	Not carried out	Liver	Multiple	CS, 38-39 GW	Conservative treatment (observation)	No interventional treatment applied
Guaman et al. (17)	Case 1	Bolivia	30 yo	3	Abdominal distension, early satiety, abdominal discomfort, respiratory difficulty, lumbar pain	Obstetric U/S, abdominopelvic CT	Adnexa	1	CS, 39 GW	Surgery	3
Schandiz et al. (18)	Case 1	Norway	33 yo	3	Asymptomatic/incidental finding	U/S	Right iliac fossa	1	CS	Conservative treatment	Postpartum
Brezeanu et al. (19)	Case 1	Romania	15 yo	2	Abdominal pain, pelvic pain, fever	Laboratory workup, urine culture, US	Adnexa	1	CS, 38 GW	Surgery	2
Mazid and De Kock (20)	Case 1	Australia	29 yo	2	Asymptomatic/incidental finding	Serology (other), US, serology Ech., spleen MRI	Spleen	1	Vaginal delivery, 39 GW	Conservative treatment	Postpartum
Gul et al. (13)	Case 1	UAE	22 yo	3	Abdominal pain, RUQ pain, gastrointestinal symptomatology	U/S, abdominal MRI, serology Ech., serology (other), laboratory workup	Liver	Multiple	CS, 38 GW	Conservative treatment (observation)	No interventional treatment applied
Segura-Gago et al. (21)	Case 1	Peru	19 yo	3	Abdominal pain, sensation/palpation of mass in LUQ, respiratory difficulty	Laboratory workup, U/S, serology (other)	Spleen	1	CS, 37 GW	Caesarean section at presentation & surgery	During CS
Li et al. (22)	Case 1	Tibet	19 yo	3	Obstetric complications	U/S, laboratory workup	Pelvis, liver	Multiple	Vaginal delivery, 38 GW	Cyst aspiration under U/S guidance	3
Thomson et al. (15)	Case 1	South Africa	41 yo	3	Left flank fullness, left shoulder pain	U/S, IHA	Spleen	Multiple	CS, 36 GW	Conservative treatment	Postpartum
Tarafdar et al. (23)	Case 1	Iran	23 yo	1	RUQ pain	U/S, laboratory workup	Adnexa	Multiple	Ongoing normal pregnancy	Surgery	1
Çankaya and Yeşilyurt (24)	Case 1	Turkey	23 yo	3	Abdominal pain	Laboratory workup, MRI	Liver	2	CS, 35 GW	Conservative treatment	During CS
Bhirud et al. (25)	Case 1	India	36 yo	1	Flank pain, abdominal pain, genitourinary symptomatology	U/S, serology Ech., laboratory workup	Kidney	1	Termination of pregnancy	Conservative treatment	After pregnancy termination
Syed and Syed (26)	Case 1	USA	40 yo	1	Asymptomatic/incidental finding	CTPA, laboratory workup, U/S, serology Ech.	Liver	1	N/A	Conservative treatment	Postpartum
Pencovich et al. (14)	Case 1	Israel	29 yo	2	Weakness, gastrointestinal symptomatology, abdominal discomfort	Laboratory workup, U/S, MRI, serology Ech.	Liver	Multiple	Vaginal delivery, 38 GW	Surgery	2
Tekin et al. (27)	Case 1	Turkey	25 yo	2	Asymptomatic/incidental finding	U/S, laboratory workup, serology Ech., abdominal and thoracic MRI	Liver, pelvis	3	Vaginal delivery, 39 GW	Surgery	2
Bakdik et al. (28)	Case 1	Turkey	37 yo	1	Asymptomatic/incidental finding	U/S, laboratory workup, IHA, serology Ech.	Liver, spleen	8	Delivery, 38GW	Cyst aspiration under U/S guidance	1
Lazaroiu et al. (29)	Case 1	Romania	29 yo	3	Jaundice, abdominal pain	laboratory workup, U/S, ERCP	Gallbladder	1	CS, 33 GW	Caesarean section at presentation & surgery	During CS
Ware et al. (30)	Case 1	India	37 yo	3	Abdominal pain, genitourinary symptomatology	Laboratory workup, U/S, chest x-Ray	Pelvis	1	CS, 37 GW	Caesarean section at presentation & surgery	During CS
Thakare (31)	Case 1	India	20 yo	3	Abdominal pain	U/S	Adnexa	2	CS, 38 GW	Caesarean section at presentation & surgery	During CS
Ghosh et al. (32)	Case 1	India	33 yo	3	Pruritis, RUQ pain, gastrointestinal symptomatology, fever	Laboratory workup, U/S, serology Ech., serology (other)	Liver	1	Vaginal delivery, 36 GW	Cyst aspiration under U/S guidance	3
Bhattacharyya et al. (33)	Case 1	India	28 yo	3	Abdominal pain	U/S, serology Ech.	Pelvis	Multiple	CS	Conservative treatment (antibiotics)	During CS
Thomspon et al. (34)	Case 1	UK	N/A	1	Abdominal pain, fever	U/S	Liver	1	CS, 38 GW	Cyst aspiration under U/S guidance	3
Tyagi et al. (35)	Case 1	India	22 yo	2	Asymptomatic/incidental finding	U/S, pelvic MRI, IHA, laboratory workup	Pelvis, right subphrenic region, right lumbar region	4	CS, 38 GW	Conservative treatment (antibiotics)	During CS
Pallua et al. (36)	Case 1	Austria	29 yo	2	Flank pain	U/S, MRI	Liver	3	CS, 34 GW	Conservative treatment (antibiotics)	Postpartum
Kanagal and Hanumanalu (37)	Case 1	India	18 yo	2	Abdominal pain, genitourinary symptomatology	Laboratory workup, U/S, surgery	Urinary bladder	1	Ongoing normal pregnancy	Surgery	2
Poiat et al. (38)	Case 1	Turkey	26 yo	2	RUQ pain, gastrointestinal symptomatology	U/S, laboratory workup, IHA	Liver	1	Vaginal delivery	Surgery	3
Kilinc and Arican (39)	Case 1	Turkey	27 yo	3	High blood pressure	laboratory workup, urinalysis, U/S	Uterus	1	CS, 33 GW	Caesarean section at presentation & surgery	During CS
Robertson et al. (40)	Case 1	South Africa	16 yo	3	Obstetric complications, gastrointestinal symptomatology	U/S	Liver	1	Vaginal delivery, 36 GW, death (patient)	Conservative treatment (antibiotics)	Postpartum
Iyilikci et al. (41)	Case 1	Turkey	38 yo	2	Asymptomatic/incidental finding	U/S, MRI, laboratory workup	Liver	1	Vaginal delivery, 37 GW	Cyst aspiration under U/S guidance	2
Sahin et al. (42)	Case 1	Turkey	29 yo	2	RUQ pain	U/S, MRI, IHA	Liver, omentum, adnexa	1	Vaginal delivery, 40 GW	Surgery	2
Brahmi et al. (43)	Case 1	Tunisia	24 yo	2	Gastrointesinal symptomatology, neurologic symptoms	ABGs, laboratory workup, U/S	Liver	1	Spontaneous miscarriage after 12 h	Miscarriage & surgery	Treatment after abortion
Can et al. (44)	Case 1	Turkey	32 yo	2	Sensation/palpation of mass in LUQ	U/S, MRI	Spleen, liver	2	Vaginal delivery, 39 GW	Surgery	2
Montes et al. (45)	Case 1	USA	26 yo	1	RUQ pain	Laboratory workup, U/S, IHA (after surgery)	Liver, right iliac fossa, greater omentum	2	7 years boy-minimally impaired writing and reading skills	Surgery	1
Srour and Sayfan (46)	Case 1	Israel	27 yo	1	Abdominal mass	Laboratory workup, U/S	Spleen, liver	1	Termination of pregnancy	Surgery (after pregnancy termination)	1
Abu-Eshy and Ali (47)	Case 1	Saudi Arabia	37 yo	3	RUQ pain, gastrointestinal symptomatology	Laboratory workup, U/S	Liver	1	Delivery	Surgery	3
Ustünsöz et al. (48)	Case 1	Turkey	26 yo	2	RUQ pain, gastrointestinal symptomatology	Serology Ech., laboratory workup	Liver	Multiple	Delivery	Cyst aspiration under U/S guidance	2
Zorlu et al. (49)	Case 1	Turkey	27 yo	3	Obstetric complications	U/S	Retroperitoneal	1	CS	Caesarean section at presentation & surgery	During CS
van Vliet et al. (50)	Case 1	Netherlands	20 yo	3	Obstetric complications	U/S, laboratory workup, serology Ech., MRI, chest x-ray	Liver	3	Vaginal delivery, 39 GW	Conservative treatment (antibiotics)	No interventional treatment
Golaszewski et al. (51)	Case 1	Austria	20 yo	2	Gastrointestinal symptomatology, RUQ pain	Laboratory workup, U/S, IHA, MRI, ERCP	Liver	1	Vaginal delivery, 33 GW, death (baby)	Surgery	2
Dhar et al. (52)	Case 1	Turkey	28 yo	3	Obstetric complications	Only clinical examination	Pelvis	1	N/A	Caesarean section at presentation & surgery	During CS
Crow et al. (53)	Case 1	USA	25 yo	2	Pruritis, RUQ pain, gastrointestinal symptomatology, fever	Laboratory workup, chest x-ray, hepititis profile, U/S, serology Ech., serology (other)	Liver	1	Vaginal delivery	Surgery	2
Vander Weiden (54)	Case 1	Netherlands	21 yo	3	Pruritis, jaundice	laboratory workup, serology (other), serology Ech., parasitologic examination of stools, U/S	Liver	1	Vaginal delivery, 37 GW	N/A	N/A
Shanbhag et al. (55)	Case 1	India	32 yo	3	Obstetric complications	N/A	Pelvis	1	CS	Surgery (evacuation)	During CS

yo, years old; U/S, ultrasound; Ech., echinococcus; CS, caesarean section; GW, gestational week; MRI, magnetic resonance imaging; N/A, not available; CT, computed tomography; UTI, urinary tract infection; RUQ, right upper quadrant; IHA, indirect haemagglutination assay; CTPA, computed tomography pulmonary angiogram; PAIR, puncture aspiration instillation re-injection; h, hours; LUQ, left upper quadrant; ERCP, endoscopic retrograde cholangio-pancreatography.

**Table 2:** Characteristics of the included studies (case series).

Author, year	Case number	Age	Trimester of diagnosis	Presenting symptoms	Diagnostic modality	Site of cysts	Number of cysts	Pregnancy outcome	Therapeutic plan in pregnancy	Trimester of management
Farès et al. (56) India	Case 1	30 yo	1	Fever, RUQ pain	Laboratory workup, U/S	Liver	1	Spontaneous abortion, 10 GW	Surgery	1
	Case 2	24 yo	2	Fever, RUQ pain	Laboratory workup, U/S	Liver	1	N/A	Surgery	N/A
	Case 3	29 yo	2	Fever, RUQ pain	Laboratory workup, U/S	Liver	2	N/A	Surgery	N/A
	Case 4	23 yo	2	Fever, RUQ pain	Laboratory workup, U/S	Liver	2	N/A	Surgery	N/A
	Case 5	22 yo	2	Fever, RUQ pain	Laboratory workup, U/S, MRI liver	Liver	2	N/A	Surgery	N/A
	Case 6	33 yo	2	RUQ pain	Laboratory workup, U/S	Liver	2	N/A	Surgery	N/A
	Case 7	24 yo	2	Fever, RUQ pain	Laboratory workup, U/S	Liver	1	N/A	Surgery	N/A
	Case 8	24 yo	2	Fever, RUQ pain	Laboratory workup, U/S	Liver	2	N/A	Surgery	N/A
Sahin et al. (57) Turkey	Case 1	N/A	N/A	Abdominal pain	N/A	N/A	N/A	CS, 39 GW	Conservative treatment (antibiotics)	N/A
	Case 2	N/A	N/A	Abdominal pain, jaundice	N/A	N/A	N/A	CS, 30 GW	Surgery	N/A
	Case 3	N/A	N/A	Asymptomatic/incidental finding	N/A	N/A	N/A	CS, 37 GW	Conservative treatment (antibiotics)	N/A
	Case 4	N/A	N/A	Abdominal pain	N/A	N/A	N/A	Vaginal delivery, 38 GW	Conservative treatment (antibiotics)	N/A
	Case 5	N/A	N/A	Abdominal pain, jaundice, dyspepsia	N/A	N/A	N/A	Abortion, 16 GW	Surgery	N/A
	Case 6	N/A	N/A	Abdominal pain, abdominal mass	N/A	N/A	N/A	CS, 32 GW	Surgery	N/A
	Case 7	N/A	N/A	Abdominal pain	N/A	N/A	N/A	Vaginal delivery, 34 GW	Cyst aspiration under U/S guidance	N/A
Noori (58) Iraq	Case 1	21 yo	3	N/A	N/A	Liver	N/A	Vaginal delivery	Cyst aspiration under U/S guidance	N/A
	Case 2	33 yo	1	N/A	N/A	Liver	N/A	Vaginal delivery	Conservative treatment (observation)	N/A
	Case 3	38 yo	2	N/A	N/A	Liver	N/A	CS	Cyst aspiration under U/S guidance	N/A
	Case 4	31 yo	2	N/A	N/A	Liver	N/A	CS	Surgery	N/A
	Case 5	28 yo	3	N/A	N/A	Liver	N/A	Vaginal delivery	Conservative treatment (observation)	N/A
	Case 6	35 yo	1	N/A	N/A	Liver	N/A	CS	Cyst aspiration under U/S guidance	N/A
	Case 7	42 yo	2	N/A	N/A	Liver	N/A	Vaginal delivery	Conservative treatment (antibiotics)	N/A
	Case 8	18 yo	1	N/A	N/A	Liver	N/A	Vaginal delivery	Conservative treatment (observation)	N/A
	Case 9	36 yo	1	N/A	N/A	Liver	N/A	CS	Conservative treatment (observation)	N/A
	Case 10	25 yo	2	N/A	N/A	Liver	N/A	CS	Cyst aspiration under U/S guidance	N/A
	Case 11	34 yo	3	N/A	N/A	Liver	N/A	Vaginal delivery	Conservative treatment (antibiotics)	N/A
	Case 12	37 yo	2	N/A	N/A	Liver	N/A	Vaginal delivery	Surgery	N/A
Ünalp et al. (59) Turkey	Case 1	28 yo	1	Abdominal pain, RUQ sensitivity	Laboratory workup, U/S	Liver	1	Vaginal delivery	Surgery	1
	Case 2	31 yo	2	Asymptomatic/incidental finding	Laboratory workup, U/S, MRI	Liver	1	Vaginal delivery, 34 GW	Surgery	2
Kain and Keystone (60) Canada	Case 1	28 yo	3	RUQ pain	Chest x-ray, U/S	Liver	multiple	Healthy child	Conservative treatment (antibiotics)	No interventional treatment applied
	Case 2	34 yo	3	RUQ pain, urticaria	N/A	Liver	N/A	Healthy child	Conservative treatment (antibiotics)	No interventional treatment applied
Rahman et al. (61) Libya	Case 1	21 yo	2	Genitourinary symptomatology, abdominal pain, gastrointestinal symptomatology	N/A	Pelvis	1	N/A	Caesarean section at presentation & surgery	During CS
	Case 2	30 yo	3	Obstetric complications	N/A	Pelvis	1	N/A	Caesarean section at presentation & surgery	During CS
	Case 3	36 yo	N/A	Obstetric complications	N/A	Adnexa	1	N/A	Caesarean section at presentation & surgery	During CS

yo, years old; RUQ, right upper quadrant; U/S, Ultrasound; N/A, not available; MRI, magnetic resonance imaging; CS, caesarean section; GW, gestational week.

**Figure 2:**
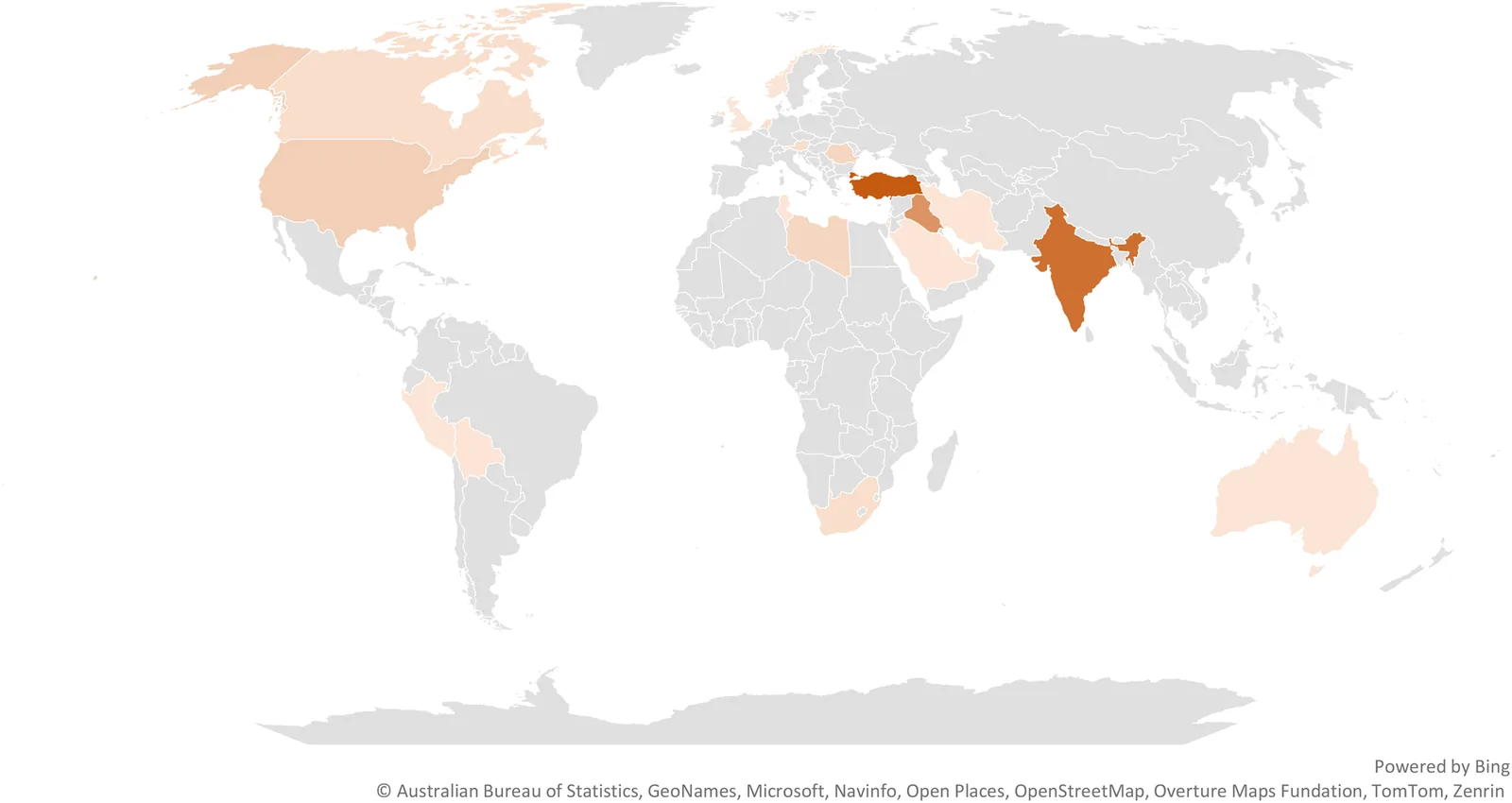
World map presenting the geographical distribution of cases included in our research.

The highest number of cases was reported in Turkey (20/79, 25.3%), India (17/79, 21.5%), and Iraq (12/79, 15.1%). Nine cases (9/79, 11.3%) were documented across Europe, with a similar number originating from North and South America (9/79, 11.3%). Nearly half of these cases were from the United States (4/79, 5%). Only six cases were reported in Africa (6/79, 7.6%), three of which were part of a single case series. One case was recorded in Australia (1/79, 1.3%). The geographical distribution of cases is presented in Figure 2.

**Figure 3:**
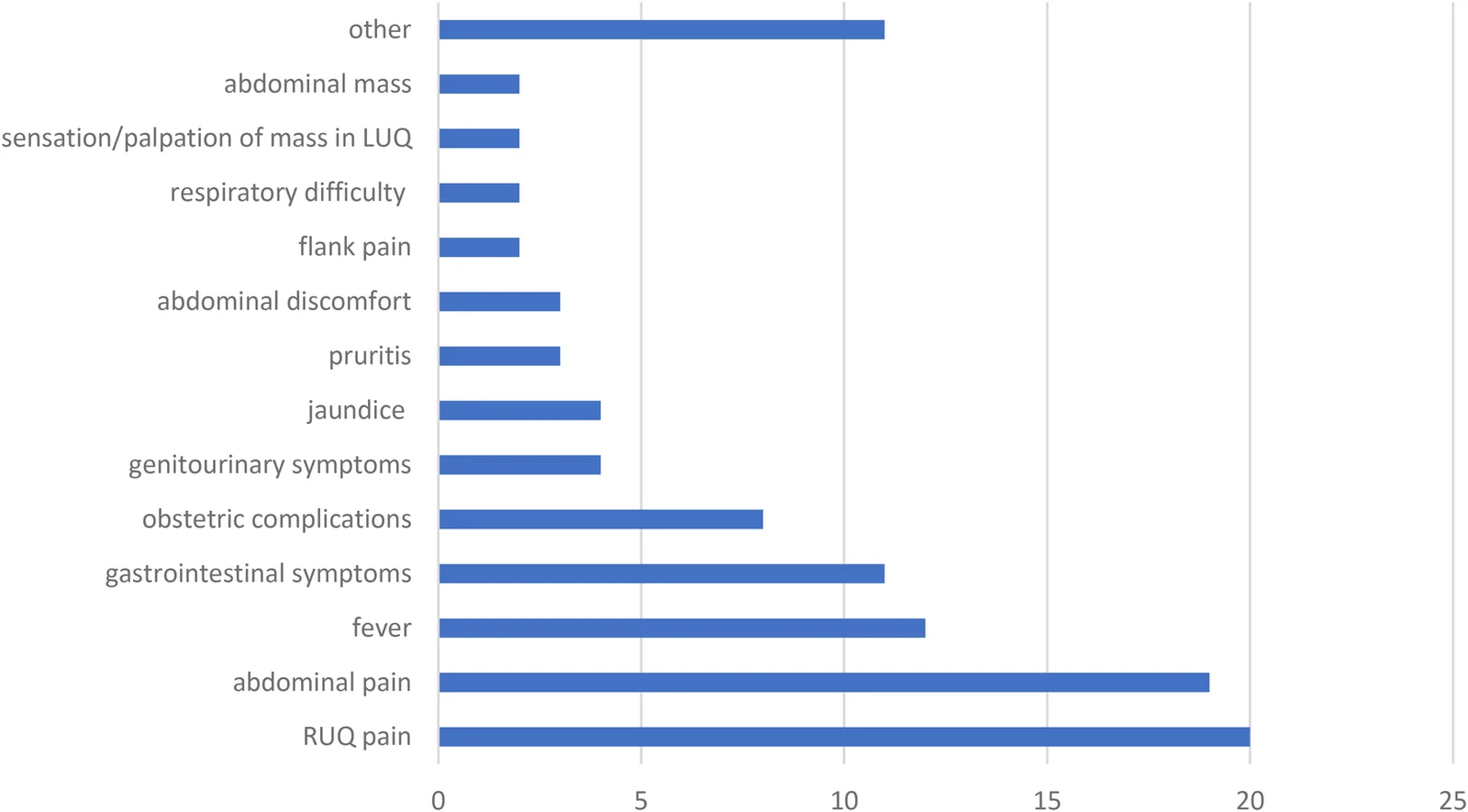
Presenting manifestations among symptomatic patients.

A total of 79 patients with abdominopelvic hydatid disease were identified. The mean age was 28 years (range 15–42). Clinical data were available for 67 cases. Eleven patients were asymptomatic (11/67, 16.4%): 10 were diagnosed incidentally during routine pregnancy visits (10/67, 14.9%), while one had been diagnosed before pregnancy and was already under surveillance (1/67, 1.5%). The remaining 56 patients were symptomatic (56/67, 83.6%). Right upper quadrant (RUQ) pain was reported in 20 patients (20/56, 35.7%), nonspecific abdominal pain in 19 (19/56, 33.9%), and fever in 12 (12/56, 21.4%). Gastrointestinal symptoms, including nausea, vomiting, or diarrhoea, were described in 11 patients (11/56, 19.6%). Obstetric complications—including abnormal foetal presentation, suspected foetal growth restriction, preterm labour, spontaneous labour—were presenting manifestations in eight cases (8/56, 14.3%). Genitourinary symptoms and jaundice were each reported in four patients (4/56, 7.1%). Abdominal discomfort and pruritus were each recorded in three cases (3/56, 5.4%). Flank pain, respiratory difficulty, an abdominal mass, and the sensation or palpation of a left upper quadrant mass were each reported in two patients (2/56, 3.6%). Other reported manifestations included abdominal distension, early satiety, pelvic pain, left flank fullness, left shoulder pain, RUQ tenderness, urticaria, hypertension, weakness, lumbar pain, and neurological symptoms (11/56, 19.6%) (Figure 3).

**Figure 4:**
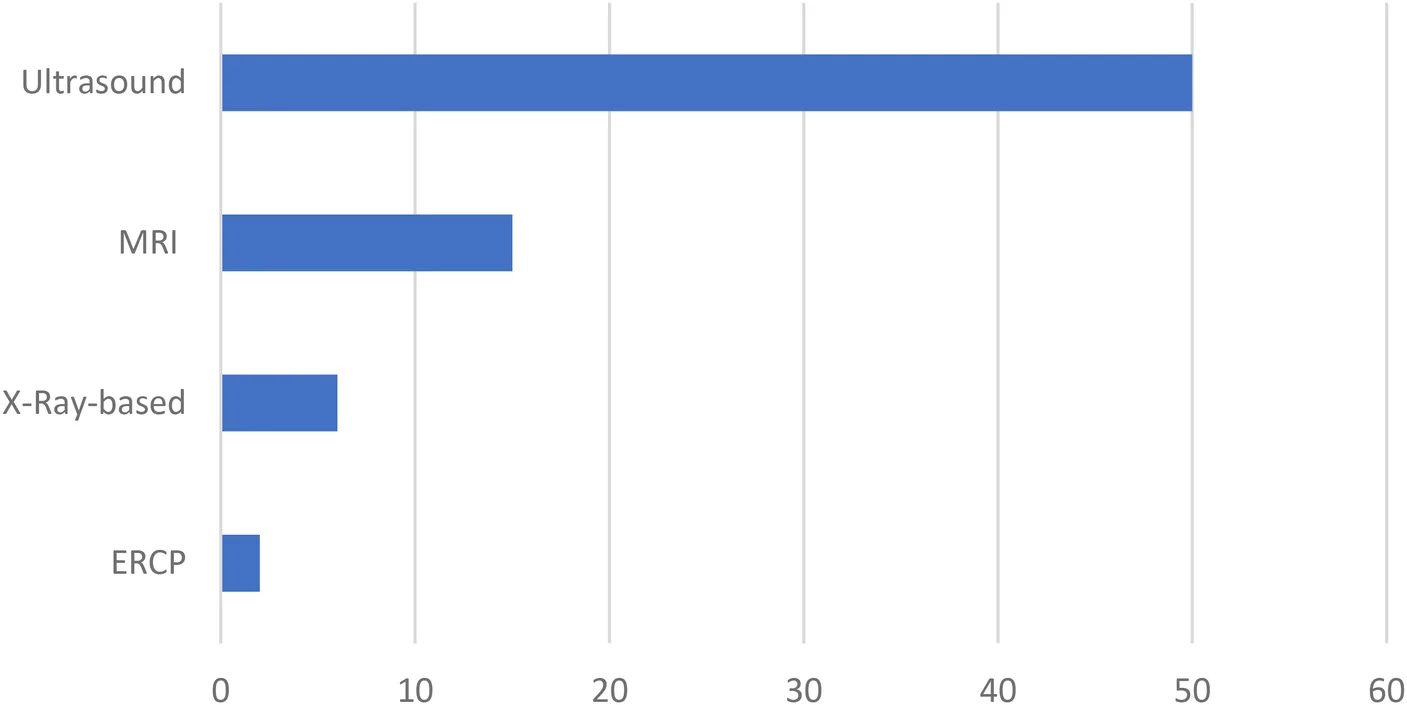
Utilised imaging modalities.

Diagnostic investigations were reported in 53 cases. Laboratory investigations were documented in 40 cases (40/53, 75.5%); results were normal in 25 (25/40, 62.5%) and at least one abnormal value was reported in 15 (15/40, 37.5%). At least one imaging modality was used in 52 cases (52/53, 98.1%). Ultrasound was performed in 50 patients (50/52, 96.1%), and MRI, either abdominal or organ-targeted, was used in 15 (15/52, 28.9%). CT, CT pulmonary angiography, or x-ray imaging was used in six cases (6/52, 11.5%), while endoscopic retrograde cholangiopancreatography was performed in two (2/52, 3.8%) (Figure 4). Ultrasound was used as a stand alone imaging modality in 31 cases (31/50, 62%). Serological testing was documented in 16 cases (16/53, 30.2%).

Echinococcosis status before the reported pregnancy was specified in only 14 of the 79 cases. Nine of these patients had a previous diagnosis of echinococcosis (9/14, 64.3%). Trimester at diagnosis was reported in 70 cases: 30 women were diagnosed in the second trimester (30/70, 42.9%), 27 in the third trimester (27/70, 38.6%), and 13 in the first trimester (13/70, 18.6%).

**Figure 5:**
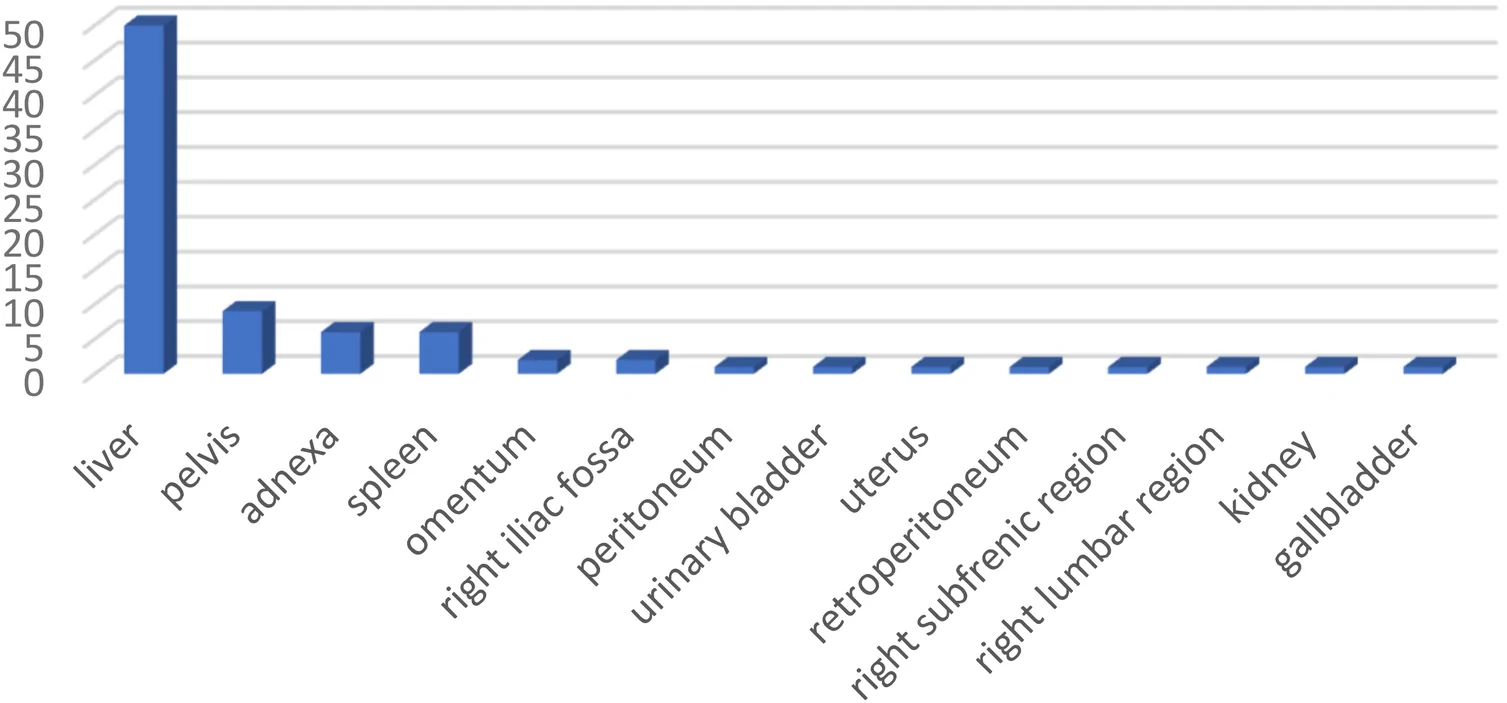
Cyst localisation within abdominopelvic cavity among pregnant women.

Cyst location was reported in 72 cases. At least one hepatic cyst was identified in 50 patients (50/72, 69.4%), while pelvic involvement was reported in nine (9/72, 12.5%). The adnexa and spleen were each involved in six patients (6/72, 8.3%), followed by the omentum and right iliac fossa in two cases each (2/72, 2.8%). Single cases involved the peritoneum, urinary bladder, uterus, retroperitoneum, right subphrenic region, right lumbar region, gallbladder, or kidney (1/72, 1.4% each) (Figure 5).

**Figure 6:**
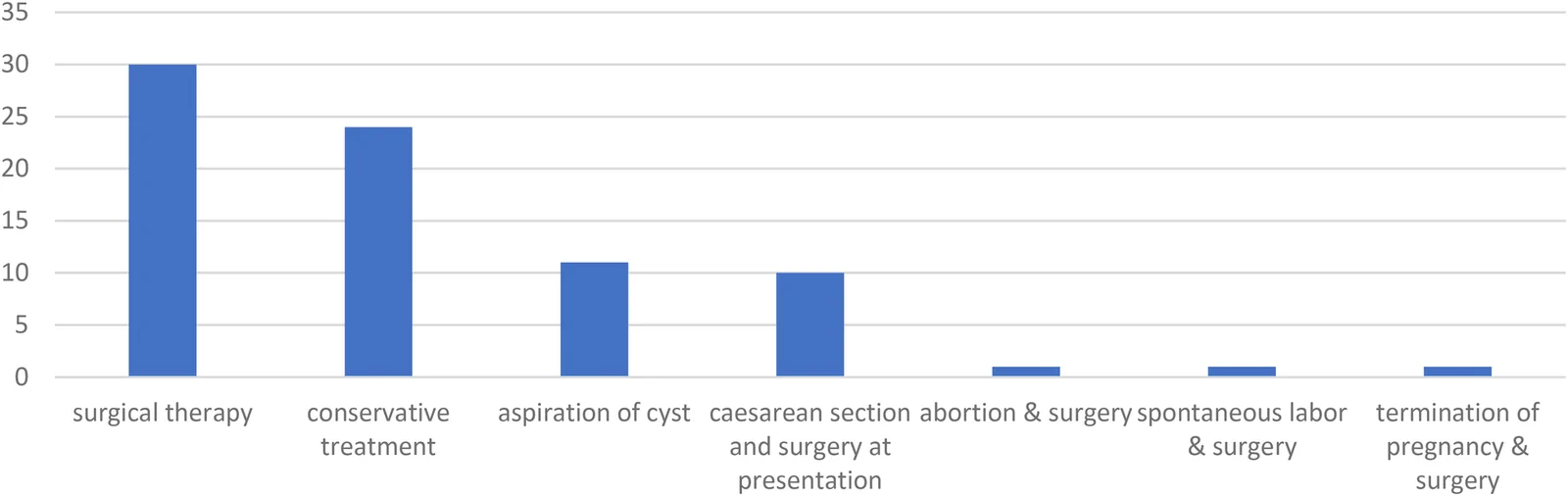
Treatment strategy across cases in our systematic review.

The therapeutic management was described in 78 cases. Surgical treatment during pregnancy was reported in 30 cases (30/78, 38.5%), while a conservative approach—defined as antibiotic therapy or a watch-and-wait strategy—was selected in 24 (24/78, 30.8%). Caesarean section performed concurrently with surgery at presentation was reported in 10 cases (10/78, 12.8%), and cyst aspiration during pregnancy in 11 (11/78, 14.1%). Miscarriage followed by surgery, termination of pregnancy followed by surgery, and spontaneous labour followed by surgery were each reported once (1/78, 1.3%) (Figure 6).

**Figure 7:**
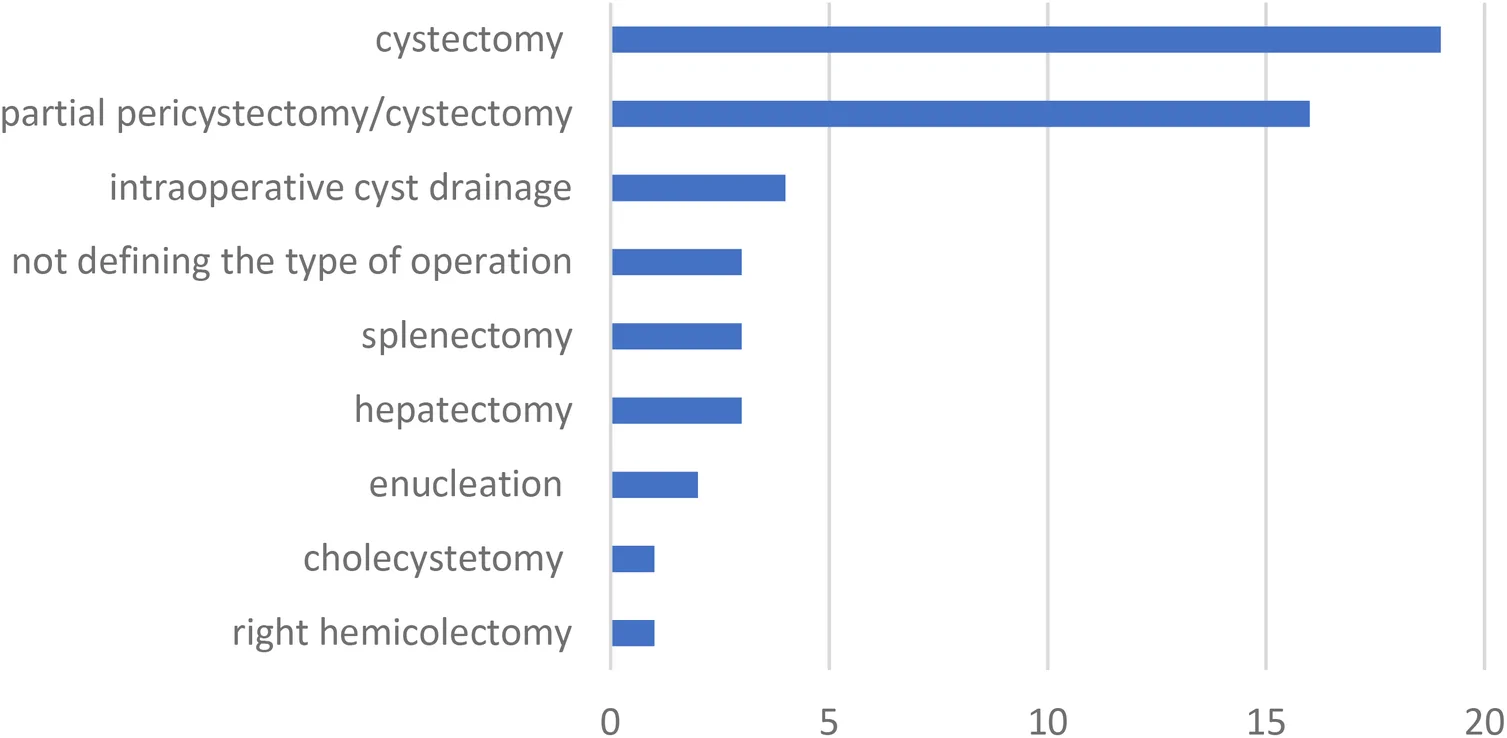
Overview of surgical treatment approaches used in the study population.

**Figure 8:**
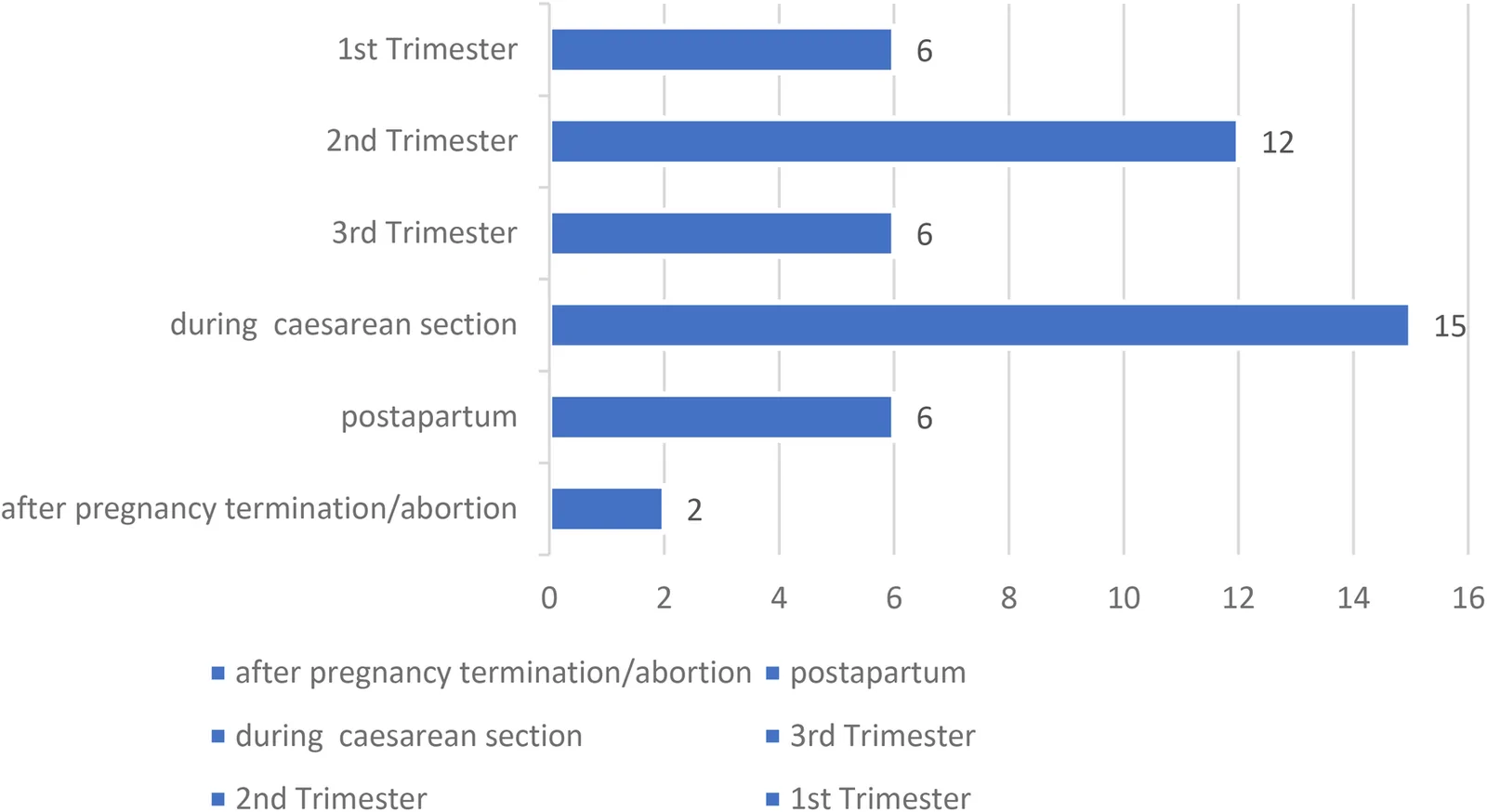
Temporal distribution of interventions in patients included in this systematic review.

The operative procedure was specified in 52 cases. Cystectomy was performed in 19 patients (19/52, 36.5%), most commonly for hepatic, pelvic, adnexal, or peritoneal cysts, with isolated procedures for splenic, renal, bladder, and uterine cysts. Partial cystectomy or pericystectomy was described in 16 patients (16/52, 30.8%), most of whom had hepatic cysts. Intraoperative cyst drainage was documented in four cases (4/52, 7.7%), while hepatectomy and splenectomy were each recorded in three (3/52, 5.8%). Enucleation was reported in two cases (2/52, 3.9%), and right hemicolectomy and cholecystectomy in one patient each (1/52, 1.9%) (Figure 7). The operative approach was reported in 46 cases: 42 procedures were open (42/46, 91.3%), while four were laparoscopic or robotic (4/46, 8.7%). The source reports generally described the procedures as technically successful; however, adverse-event reporting was incomplete in many studies. Mean cyst diameter was 11.38 cm in the surgically treated group (reported in 32 cases) and 12.3 cm in the drainage group (reported in six cases). Intervention timing was reported in 47 cases. Procedures were performed during caesarean section in 15 cases (15/47, 31.9%), during the second trimester in 12 (12/47, 25.5%), and during the first trimester, third trimester, or postpartum in six cases each (6/47, 12.8% each). Two procedures followed pregnancy termination or miscarriage (2/47, 4.3%) (Figure 8).

Benzimidazole therapy was administered to 31 patients, either as definitive, adjuvant, or neoadjuvant treatment. Therapy was initiated during pregnancy in 22 cases (22/31, 71%) and after delivery in nine (9/31, 29%). Two of the pregnancies were terminated, and in three reports further details regarding the timing of therapy were unclear.

Pregnancy outcomes were documented in 67 cases. Caesarean delivery occured in 29 cases (29/67, 43.3%), while vaginal delivery occurred in 23 (23/67, 34.3%). The mode of delivery was not specified in five cases (5/67, 7.5%), and two pregnancies were ongoing and uncomplicated at the time of reporting (2/67, 3%). Pregnancy termination occurred in two cases (2/67, 3%), and miscarriage in three (3/67, 4.5%). One neonatal death and one maternal death were reported (1/67, 1.5% each). One child was reported to have impaired neurodevelopment at seven years of age (1/67, 1.5%).

### Quality of the included studies

The quality of the studies was assessed using the JBI critical appraisal checklists (62, 63). Most case reports were rated as either good or moderate, with a perfect score achieved by only one study. The majority of the included reports fulfilled more than 60% of the quality criteria, while four reports met 50% or fewer of the criteria and were therefore classified as low-quality studies with a high risk of bias. Across the 45 case reports, the most frequently missing domain was Q7. Most case series were also classified as good or moderate, with none achieving a perfect score. One case series fulfilled less than 50% of the quality criteria and was consequently judged to be of low quality, with a high risk of bias.

## Discussion

The present systematic review aimed to examine all available evidence reporting cases of abdominopelvic hydatid disease during pregnancy. Few published cases originated from Africa, which may reflect underdiagnosis or underreporting rather than true disease rarity. Similar geographic patterns have been described in previous studies (5). Only nine patients had a documented history of echinococcosis. This may reflect incomplete reporting; any role of pregnancy-related immunological changes in disease progression or reactivation remains uncertain (64). Ultrasound was the most frequently used imaging modality and provided sufficient diagnostic information in more than half of the cases, highlighting its role as a safe and effective first-line tool for abdominal pathology during pregnancy (65). Serological testing was documented in 16 cases. Previous literature indicates that abdominal CE is primarily diagnosed using ultrasonography, with serology used as an adjunctive diagnostic tool, which is consistent with the pattern observed in the present review (66). Importantly, pregnancy limits the use of certain imaging modalities due to concerns regarding foetal radiation exposure (4). Diagnosis was most common in the second (42.9%) and third trimesters (38.6%), consistent with previous literature; however, this pattern may also reflect differences in the timing and availability of imaging (58).

The liver was the most common involvement site (50/72, 69.4%), consistent with the established CE pathophysiology (21). Other locations included the pelvis (9/72, 12.5%) and the spleen (6/72, 8.3%). Adnexal involvement, which is traditionally considered a rare site for echinococcal cysts, was identified in six cases (6/72, 8.3%) (5). Adnexal or pelvic cysts may be mistaken for other gynaecological pathology, including malignancy or endometriosis. Common presenting manifestations in the review included RUQ pain (20 patients), abdominal pain (19 patients), and obstetric complications (8 patients). The predominant obstetric complications observed were abnormal foetal presentation (4 cases) and spontaneous preterm labour (2 cases). These findings, along with abdominal pain, are consistent with obstetric complications previously described in pregnant women with symptomatic abdominopelvic CE (4). Fever and gastrointestinal symptoms were also commonly reported, whereas genitourinary manifestations occurred in only four women. Other clinical presentations were sporadic. Overall, either RUQ or nonspecific abdominal pain was documented in 39 of 56 symptomatic patients (69.6%), a higher proportion than that reported by a previous study, where abdominal pain was observed in 57.3% of the cases in the general population (67). Fever was also more frequently recorded as a presenting symptom in 12 cases (21.4%), as were gastrointestinal manifestations (19.6%), exceeding previously reported rates. Nausea and vomiting were the most frequently documented gastrointestinal symptoms in our study, with diarrhoea reported in only one patient. It remains uncertain whether these differences reflect random variation, selective reporting, or a genuinely different clinical presentation during pregnancy.

Surgical intervention was the most commonly selected therapeutic strategy (38.5%), while conservative treatment was also preferred in 24 cases. In 10 patients, surgery was scheduled to coincide with caesarean section, representing a notable combined management approach. Given the diagnostic and therapeutic complexity, multidisciplinary input may be appropriate when planning management (2). Conservative treatment may be selected to prioritise foetal safety by avoiding anaesthetic exposure and surgical stress (2). Cyst aspiration was performed in 11 cases; however, previous studies have associated aspiration techniques with higher recurrence rates and the need for additional interventions, potentially explaining their limited use as a primary strategy (68). One miscarriage and one spontaneous labour occurred before any intervention could be initiated. The most common timing for intervention was during caesarean section (31.9%), followed by the second trimester (25.5%), which is generally associated with lower risks of adverse events (14). Previous literature describes complete cyst excision as a preferred approach when feasible, while partial cystectomy, pericystectomy, or deroofing may be considered in selected cases (69). An operative procedure was described in 52 patients, irrespective of timing. Cystectomy was the most frequently employed operative technique (36.5%), followed by partial cystectomy or pericystectomy performed in 16 patients (30.8%). More radical procedures, such as hepatectomy and splenectomy, were reserved for selected cases, and a right hemicolectomy and a cholecystectomy were performed in only one patient each. Intraoperative cyst drainage (4/52, 7.7%) and enucleation (2/52, 3.9%) were less commonly utilised. In contrast, a previous systematic review and meta-analysis examining the surgical management of abdominal cystic echinococcosis in the general population reported complete cyst excision in 85.7% of cases, a markedly higher proportion compared with the 42% observed in our study (70). This apparent difference may reflect greater reliance on partial excision techniques during pregnancy, potentially to minimise operative risk (70). The predominance of open surgical techniques (91.3%) observed in our study aligns with findings from the same study, where open surgery accounted for 85.7% of procedures (70).

The use and timing of benzimidazole therapy during pregnancy remain of particular interest. Twenty-two of thirty-one patients for whom benzimidazole treatment was chosen received the first dose during gestation. The safety of benzimidazoles during pregnancy, particularly during the first trimester, remains uncertain because the available evidence is limited. A previous study showed no statistically significant increase in adverse events among pregnant individuals at early gestation receiving benzimidazoles, although no study specifically addressed first-trimester exposure (71). Previous literature suggests that perioperative benzimidazole therapy may reduce recurrence, which has been reported in up to 11% of untreated cases (14). Preterm delivery occurred in 13 of the 36 pregnancies (36.1%) for which gestational age at delivery was documented. Although CE has been implicated as a potential trigger for preterm labour, its true incidence remains insufficiently defined in the existing literature (58). Eight of these deliveries were by caesarean section, and one neonatal and one maternal death were reported in this subgroup. Notably, a previous study reported a significantly higher mortality rate among pregnant women with CE compared with the non-pregnant population (72). Among women who delivered at term (23/36, 63.9%), caesarean section and vaginal delivery were nearly equally represented (13 and 10 cases, respectively). Three miscarriages and two elective pregnancy terminations were also documented. Pregnancy loss was among the most serious outcomes reported (73). Although increased intra-abdominal pressure during vaginal delivery could theoretically increase the risk of cyst rupture and subsequent anaphylaxis, the available case reports did not provide sufficient comparative evidence to determine whether cyst size or rupture risk differed by mode of delivery (74). A cyst rupture rate of approximately 4.5% during pregnancy has been reported in previous literature (20).

### Strengths and limitations

To our knowledge, this is the first systematic review to provide a comprehensive overview of reported cases of abdominopelvic cystic echinococcosis during pregnancy. The review incorporated a systematic database search, individual patient-level data extraction, and quality appraisal using validated JBI tools. Nevertheless, the findings must be interpreted cautiously. The evidence base consisted exclusively of case reports and small case series, which are inherently susceptible to selection, reporting, and interpretation bias and cannot establish causal relationships or support reliable comparisons between management strategies. Clinical characteristics and outcomes were inconsistently reported, resulting in variable denominators across analyses. Accordingly, the proportions presented in this review should be regarded as descriptive summaries of published cases rather than estimates of disease incidence, comparative treatment effectiveness, or risk. Publication bias is also an important consideration because unusual, severe, surgically managed, or otherwise clinically noteworthy cases are more likely to be reported, while uncomplicated cases and patients managed expectantly may be underrepresented. Restriction to full-text English-language publications may have excluded relevant reports from endemic regions and may have influenced the observed geographic distribution and management patterns. Cases embedded within larger series or incompletely indexed reports may also have been missed. Formal statistical assessment of publication bias was not appropriate because the review was descriptive and included predominantly case reports and small case series. Some included reports were of limited methodological quality, and the small number of patients precluded firm conclusions regarding optimal diagnostic or management strategies. Prospective international registries using standardised definitions and reporting frameworks are needed to strengthen the evidence base.

## Conclusion

Abdominopelvic cystic echinococcosis during pregnancy is rare and may create important diagnostic and therapeutic challenges. The published literature indicates that ultrasound is the most frequently used diagnostic modality and that management is highly individualised. However, the available evidence consists predominantly of case reports and small case series and is subject to substantial selection, reporting, and publication bias. No firm conclusions can therefore be drawn regarding the optimal timing or type of intervention, use of antiparasitic therapy, or mode of delivery. Multidisciplinary assessment in centres with relevant expertise may be appropriate, and prospective international registries with standardised reporting are required to provide more reliable evidence.

## Author's note

Patients and members of the public were involved in shaping the development of this project. Their feedback informed the research question and refined the study design. We will share the study findings with participants and the broader patient community through accessible summaries and presentations. We remain committed to involving patients and the public throughout the research process to maximise its relevance, transparency and impact.
