# Supplementary material for: Abdominopelvic hydatid disease during pregnancy: a systematic review of the literature
Source: Front Glob Womens Health. 2026 Aug 27;7:1928979. doi: 10.3389/fgwh.2026.1928979 (PMC13559224; doi:10.3389/fgwh.2026.1928979)
Supplement: Supplementary file 1 [file Table1.docx]

**Supplementary material**

**Table S1.** Quality assessment of the included studies (studies with individual patient data)

| **Author, year** | **Q1** | **Q2** | **Q3** | **Q4** | **Q5** | **Q6** | **Q7** | **Q8** |
| --- | --- | --- | --- | --- | --- | --- | --- | --- |
| Steriou L, 2025 | ● | Ø | ● | ● | ● | ● | ○ | ● |
| Maheshwari P, 2025 | Ø | Ø | ● | ● | ● | ● | ○ | ● |
| Fanning Z, 2025 | ● | ● | ● | ● | Ø | ● | ○ | ● |
| Guaman MJ, 2025 | ● | ● | ● | ● | ● | ● | ○ | ● |
| Schandiz H, 2024 | Ø | ● | ● | ● | ● | ● | ○ | ● |
| Brezeanu AM, 2024 | ● | Ø | ● | ● | ● | ● | ● | ● |
| Mazid A, 2024 | ● | ● | Ø | ● | ● | ● | ● | ● |
| Gul M, 2023 | ● | Ø | ● | ● | ○ | ○ | ○ | ● |
| Segura-Gago GP, 2022 | ● | ● | ● | ● | ● | ○ | ○ | ● |
| Li P, 2022 | Ø | Ø | ● | ● | Ø | ● | ○ | ● |
| Thomson KF, 2022 | ● | Ø | ● | ● | ○ | ○ | ○ | ● |
| Tarafdar A, 2022 | ● | ● | ● | ● | ● | ● | ○ | ● |
| Çankaya BY, 2021 | Ø | Ø | Ø | ● | Ø | ○ | ○ | ● |
| Bhirud DP, 2020 | ● | Ø | ● | ● | ● | ● | ○ | ● |
| Sayed A, 2020 | ○ | ○ | ● | ● | Ø | ○ | ○ | ● |
| Pencovich N,2019 | ● | Ø | ● | ● | ● | ● | **○** | ● |
| Tekin AF, 2018 | ● | Ø | ● | ● | ● | ● | ○ | ● |
| Bakdik S, 2018 | ● | Ø | ● | ● | ● | ● | ● | ● |
| Lazaroiu AM, 2015 | ● | ○ | ● | ● | ● | ● | ● | ● |
| Ware S, 2015 | ● | Ø | ● | ● | ● | ○ | ○ | ● |
| Thakare P, 2015 | ○ | ○ | Ø | ● | ● | ● | ● | ● |
| Ghosh JK, 2014 | ● | Ø | ● | ● | Ø | ● | ○ | ● |
| Bhattacharyya SK, 2013 | ● | Ø | ● | ● | ● | ○ | ○ | ● |
| Thomson A, 2012 | ○ | ○ | ○ | Ø | Ø | ● | ○ | ● |
| Tyagi S, 2012 | Ø | Ø | ● | ● | ● | ● | ● | ● |
| Pallua K, 2010 | Ø | Ø | ● | ● | ● | ● | ● | ● |
| Kanagal DV, 2010 | ● | Ø | ● | ● | ● | ● | ○ | ● |
| Polat C, 2007 | ● | Ø | ● | ● | ● | ● | ● | ● |
| Kilinc N, 2007 | ● | ○ | ● | ● | ● | Ø | ○ | ● |
| Robertson M, 2006 | ○ | ○ | ● | ● | ● | ● | ● | ● |
| Iyilikci L, 2006 | ● | Ø | ● | ● | ● | ○ | ○ | ● |
| Sahin E, 2005 | Ø | Ø | ● | ● | ● | ● | ○ | ● |
| Brahmi N, 2004 | ● | ○ | ● | ● | ○ | ○ | ○ | ● |
| Can D, 2003 | Ø | Ø | ● | ● | Ø | Ø | ○ | ● |
| Montes H, 2002 | ● | Ø | ● | ● | ● | ● | Ø | ● |
| Srour SF, 2001 | ● | ● | ● | ● | ● | ● | ● | ● |
| Abu-Eshy S, 1999 | ● | Ø | ● | ● | ● | ● | ○ | ● |
| Ustünsöz B, 1999 | ● | Ø | ● | ● | ● | ● | ○ | ● |
| Zorlu CG, 1996 | Ø | Ø | ● | ● | ● | ● | ○ | ● |
| van Vliet W, 1995 | ● | ● | ● | ● | ● | ● | ○ | ● |
| Golaszewski T, 1995 | ● | Ø | ● | ● | ● | ● | ● | ● |
| Dhar KK, 1993 | Ø | Ø | ● | ● | ● | ● | ○ | ● |
| Crow JP, 1990 | ● | Ø | ● | ● | ● | ● | ○ | ● |
| Vander Weiden, 1989 | ● | ● | ● | ● | Ø | ○ | ○ | ○ |
| Shanbhag AM, 1974 | Ø | ● | ● | ● | ● | ○ | ○ | ● |

Q1: Patient demographics; Q2: Medical History; Q3:	Presentation; Q4: Diagnostic tests; Q5: Interventions; Q6: Post-intervention condition; Q7: Adverse events; Q8: Takeaway lessons ● = 1; ○ = No; Ø =Unclear, N/A= Not applicable

**Table S2.** Quality assessment of the included studies (Case series studies with individual patient data)

| **Author, year** | **Q1** | **Q2** | **Q3** | **Q4** | **Q5** | **Q6** | **Q7** | **Q8** | **Q9** | **Q10** |  |  |  |
| --- | --- | --- | --- | --- | --- | --- | --- | --- | --- | --- | --- | --- | --- |
| Farès M, 2023 | ● | ● | ● | ● | ● | ○ | ● | ● | Ø | N/A |  |  |  |
| Sahin O, 2021 | ○ | ● | ● | ● | ○ | ○ | ● | Ø | ○ | N/A |  |  |  |
| Noori IF, 2021 | ○ | ● | ● | ● | ● | ○ | ○ | ● | ● | N/A |  |  |  |
| Ünalp H, 2008 | ○ | Ø | ● | ○ | Ø | ● | ● | ● | ● | N/A |  |  |  |
| Kain KC, 1988 | Ø | ● | ● | ○ | ○ | ● | Ø | ● | ○ | N/A |  |  |  |
| Rahman MS, 1982 | ○ | ○ | ● | ● | ● | Ø | ○ | Ø | ○ | N/A |  |  |  |

Q1: Inclusion criteria ; Q2: Condition measure; Q3: identification of the condition; Q4: consecutive inclusion; Q5: complete inclusion; Q6: demographics; Q7: clinical information; Q8: outcomes or follow up; Q9: presenting site(s)/clinic(s); Q10: statistical analysis ● = 1; ○ = No; Ø =Unclear, N/A= Not applicable
